# Isolated Crohn’s disease of the esophagus with esophago-mediastinal fistula formation

**DOI:** 10.1186/1477-7819-10-208

**Published:** 2012-10-03

**Authors:** Wuping Wang, Yunfeng Ni, Changkang Ke, Qingshu Cheng, Qiang Lu, Xiaofei Li

**Affiliations:** 1 Department of Thoracic Surgery, Tangdu Hospital, The Fourth Military Medical University, Xi’an, PR China

**Keywords:** Crohn’s disease of esophagus, Esophago-mediastinal fistula, Stricture, Noncaseating granulomas

## Abstract

Isolated Crohn’s disease of the esophagus is rare, and accurate diagnosis and treatment in its early course are difficult. Most cases are often found very late, when severe strictures or other complications have occurred. We report the case of a male 60-year-old patient with complaints of progressive dysphagia for more than two months and the sudden appearance of heartburn for seven consecutive days. Clinical examination revealed severe esophageal stricture with a suspected fistula and mediastinitis. The patient received a successful esophagectomy. The resected specimen and pathological results confirmed a deep linear ulcer, chronic and noncaseating granulomatous inflammation, as well as a circular stricture of the esophagus with fistula into the mediastinum due to isolated esophageal Crohn’s disease.

## Background

Esophageal Crohn’s disease (CD) is rare, with an adult prevalence of 0.2% to 3% in patients with coexisting ileocolonic disease. Very few cases of isolated esophageal involvement are reported [1,2]. An accurate diagnosis and treatment is often made rather late in its course, due to the unusual presentation of isolated esophageal CD, particularly if patients present with complications such as severe dysphagia secondary to stricture or perforation and/or fistula formation, which could require surgical intervention [3-5]. Here, we present a case of esophago-mediastinal fistula in isolated esophageal CD, who underwent esophagectomy for severe esophageal stricture with fistula into the mediastinum.

## Case presentation

**Figure 1:**
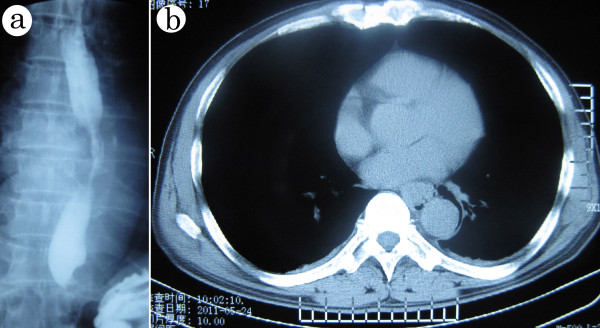
**Imaging examinations of patients.** (**a**) Irregular 6-cm narrowing of the middle esophagus in barium-swallow examination. (**b**). Thickened middle esophageal wall, with pneumomediastinum and bilateral effusion in CT scan. CT, computed tomography.

**Figure 2:**
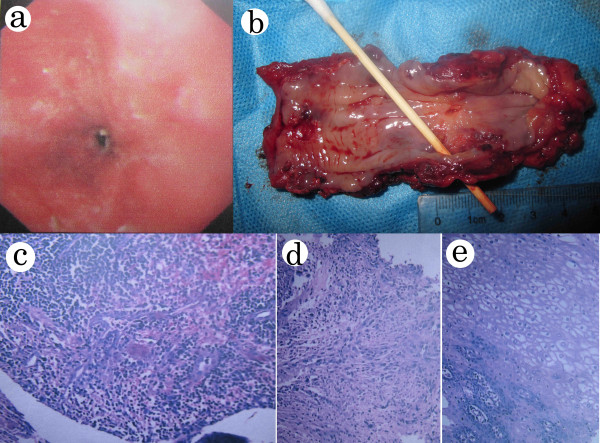
**Results of endoscopic examination, postoperative gross specimen and pathologic examination.** (**a**) Endoscopy revealed circular stricture of esophagus at 25 cm from the incisor teeth. (**b**) Gross specimen of resected esophagus showed a deep, linear, longitudinal and transmural ulceration (20 × 15 mm), which perforated into the mediastinum. All layers of the esophagus were thickened, but the mucosa was smooth. (**c**) Pathology examination showed chronic inflammation of ulcerative esophagus. HE, 100×. (**d**) Pathology showed noncaseating granulomatous of the submucosa tissue. HE, 100×. (**e**) Pathology showed squamous cell proliferation of the esophageal mucosa. HE, 300×.

A 60-year-old man was admitted to our hospital with complaints of progressive dysphagia for more than two months and the sudden appearance of heartburn for seven days. No episodes of fever, cough, diarrhea or abdominal pain were reported. On admission, an esophagogram demonstrated a 6-cm long irregular narrowing of the middle esophagus without a communicating fistulous tract (Figure 1a). Esophagoscopy revealed the presence of a circular stricture of the esophagus at 25 cm from the incisor teeth, with only a pediatric gastroscope able to pass the stricture (Figure 2a). His stomach and duodenum appeared normal. However, a thoracic computed tomography scan revealed a thickened middle esophageal wall, with pneumomediastinum, which indicated the existence of an esophago-mediastinal fistula (Figure 1b).

Although repeated biopsies confirmed only nonspecific inflammation, thoractomy was performed immediately for a suspected esophageal fistula.

The surgery revealed that the soft tissue surrounding the esophagus was edematous, with pneumomediastinum, but no pleural effusions. The local inflammation was limited without chest effusion. The patient finally received esophagectomy.

The resected specimen showed a circular esophageal stricture with a deep, linear, longitudinal and transmural ulceration (20 × 15 mm), which perforated into the mediastinum (Figure 2b). The postoperative pathology confirmed a chronic, noncaseating granulomatous inflammation with lymphocyte cell infiltration and squamous cell proliferation (Figure 2c, d, f). No abnormalities of other digestive tract sites including terminal ileum were found through further endoscopy. The patient was diagnosed with isolated esophageal CD. His postoperative evolution was uneventful. At one and a half years follow-up the patient was eating a normal diet. Examination revealed no recurrence of CD.

## Discussion

CD is a chronic inflammatory disease of unknown etiology characterized by chronic, granulomatous, segmental transmural inflammation that may occur in any part of the alimentary tract from mouth to anus. In the human upper digestive tract, the esophagus is the least common segment involved in CD [1,2]. Almost all the esophageal CD reported in the literature has coexisted with CD at other sites, such as the ileum, rectus and colorectum [1,3,4]. Here, we report the case of a patient with isolated esophageal CD in need of surgical intervention.

In general, CD of the esophagus is not difficult to diagnose in cases in which other segments of the digestive tract are also involved, or in patients with a prior history of CD. Typical endoscopic and radiographic appearance combined with histological examination contribute to a definitive diagnosis [2,5].

Previous literature reviews, demonstrate the common endoscopy findings for esophageal CD, which include aphthous ulcers, deep ulcerations, nodularity, erythema, pseudopolyps, stricture and fistula [3,6], and that the typical histological changes of CD in the esophagus are noncaseating granulomas [4]. Therefore, once a patient presents with the above characteristics, the diagnosis may be feasible. However, the granulomas are not always obvious. To the best of our knowledge, the typical granulomas are more likely located in the deep submucosa and lamina muscularis, difficult to obtain in effective biopsy, and the noncaseating granulomas occur only in less than 25% of reported cases [1,6,7]. On the other hand, most cases have presented with only nonspecific endoscopy findings as well as inflammation in pathology, which can be considered compatible with CD in highly-suspect individuals. Because of these conditions, for certain patients in whom the esophagus alone is involved, the diagnosis of esophageal CD is achieved only after definite exclusion of such possible causes as reflux esophagitis, viral esophagus, carcinoma, Behcet’s syndrome, epidermolysis bullosa acquisita, drug-induced ulcer, intramural diverticulosis, and so on. [3]. In our case, the biopsy also revealed that only a nonspecific change caused difficulties in the diagnosis of esophageal CD before surgery. The resected specimen finally confirmed an accurate diagnosis. As has been shown in the literature, three different stages for lesions in esophageal CD have been established. In the first phase, inflammatory lesions, erosions and elongated ulcerations appear from edema of the esophageal mucosa. The patient has no significant symptoms of dysphagia or odynophagia [8]. In the second phase, constrictions of the esophagus and stenosis appear usually on a section of over 1 cm, and the formation of mucosal bridges is observed [8].

In the third stage, the patient presents with progressive dysphagia and odynophagia with vomiting. At this stage, a few patients can be cured through medication and many patients with persistent dysphagia caused by stricture need esophageal dilatation. This is the end stage of the disease. Severe complications such as severe stricture and fistula occur, and many patients with persistent dysphagia, caused by a stricture, will require esophageal dilatation. Recurrent mediastinal inflammation/abscess or pneumomediastinum, could be signs of fistula formation. Barium swallow, computed tomography and esophagoscopy are used to define fistulae anatomy. Once esophageal fistula is confirmed, surgery is required to prevent further complications [9,10].

Previous studies have reported fistula formation between the pleural cavity, bronchus, esophageal wall, and even the stomach [3,11-13]. There was one case report describing successful CD-associated esophagobronchial fistula closure after therapy with infliximab, while another case report describes successful treatment with injection of a liquid polymer [14,15]. However, we did not identify any reports of CD associated esophago-mediastinal fistula. Our case reports an esophageal fistula to the mediastinum due to the end stage of isolated esophageal CD. Esophagectomy with gastric pull-through was performed in a timely fashion, when the mediastinal infection was limited.

## Conclusions

In conclusion, the diagnosis and treatment of rare esophageal CD cases is difficult. Its diagnosis should be based on clinical, endoscopic and histologic evidence, while medication, endoscopic dilation and surgical techniques might be required at different stages of the disease process. Although esophago-mediastinal fistula is a rare manifestation of CD, fistula formation should be entertained if heartburn and pneumomediastinum occur, even if it is not obvious. Our case highlights features of the end stage of the disease, revealing that early discovery along with surgical intervention may give promising results.

## Consent

Written informed consent was obtained from the patient for publication of this case report and any accompanying images. A copy of the written consent is available for review by the Editor-in-Chief of this journal.

## Abbreviations

CD: Crohn’s disease.

## Competing interests

The authors declare that they have no competing interests.

## Authors’ contributions

WW developed the study concept and design. YN acquired, analyzed and interpreted the data. CK critically revised the manuscript for important intellectual content. QC provided technical and material support. QL and XL drafted the manuscript. All authors read and approved the final manuscript.

## Authors’ information

Wuping Wang, master’s degree, Superintendent of the institution, surgeon; Yunfeng Ni, doctor’s degree, surgeon; Changkang Ke, master’s degree, surgeon; Xiaofei Li, doctor’s degree, surgeon; Qiang Lu, doctor’s degree, surgeon; Qingshu Cheng, doctor’s degree, surgeon.
